# Relationships Between Self-Rated Health at Three Time Points: Past, Present, Future

**DOI:** 10.3389/fpsyg.2021.763158

**Published:** 2022-01-13

**Authors:** Andreas Hinz, Michael Friedrich, Tobias Luck, Steffi G. Riedel-Heller, Anja Mehnert-Theuerkauf, Katja Petrowski

**Affiliations:** ^1^Department of Medical Psychology and Medical Sociology, Leipzig University, Leipzig, Germany; ^2^Faculty of Applied Social Sciences, University of Applied Sciences Erfurt, Erfurt, Germany; ^3^Institute of Social Medicine, Occupational Health and Public Health, Leipzig University, Leipzig, Germany; ^4^Department of Medical Psychology and Medical Sociology, University Medical Center of the Johannes Gutenberg University Mainz, Mainz, Germany; ^5^Department of Internal Medicine III, University Hospital Carl Gustav Carus Dresden, Dresden, Germany

**Keywords:** retrospective assessment, health, recall bias, optimism, response shift bias

## Abstract

**Background:** Multiple studies have shown that people who have experienced a serious health problem such as an injury tend to overrate the quality of health they had before that event. The main objective of this study was to test whether the phenomenon of respondents overrating their past health can also be observed in people from the general population. A second aim was to test whether habitual optimism is indeed focused on events in the future.

**Method:** A representatively selected community sample from Leipzig, Germany (*n* = 2282, age range: 40–75 years) was examined. Respondents were asked to assess their current health, their past health (5 years before), and their expected future health (in 5 years) on a 0–100 scale. In addition, the study participants completed several questionnaires on specific aspects of physical and mental health.

**Results:** Respondents of all age groups assessed their health as having been better in the past than it was at present. Moreover, they also assessed their earlier state of health more positively than people 5 years younger did their current state. Habitual optimism was associated with respondents having more positive expectations of how healthy they will be in 5 years time (*r* = 0.37), but the correlation with their assessments of their current health was nearly as high (*r* = 0.36).

**Conclusion:** Highly positive scores of retrospectively assessed health among people who have experienced a health problem cannot totally be accounted for by a response to that health problem.

## Introduction

Health-related quality of life (QoL) is an important outcome criterion in clinical research and practice. In contrast to objective indicators of health, the focus of QoL is on the subjective experience of health-related issues (Guyatt et al., 1993). Multiple questionnaires have been developed for measuring subjectively experienced health (Tengs and Wallace, 2000). For epidemiological studies, a simple one-item assessment of the subjectively experienced overall health state has proved to be a suitable criterion. While health quality is often rated using five response options (e.g., poor,…, excellent), it is also appropriate to use a 0–100 visual analog scale (VAS) to quantify respondents’ self-assessed health. This VAS is also included in the QoL instrument EQ-5D (Brooks, 1996).

It would be optimal when researchers or clinicians intend to assess changes in QoL or in subjectively experienced health resulting from an event such as injury if health data that predates the event were available to compare with post-event data. Since such data are not available in most cases, however, patients can instead be asked to retrospectively assess how healthy they were before the event. A comparison can then be made between their present quality of health and their retrospectively assessed pre-event health. The difference between the two measurements quantifies the effect of the event (Scholten et al., 2017).

The drawback of this method is that people tend to overrate their health in the past. One typical example is a Dutch study (Graaf et al., 2019) in which a total of 596 injured patients rated their pre-injury health as having been significantly better than that seen in Dutch normative data. This means that the difference between the respondents’ retrospectively assessed pre-injury health and their post-injury health probably systematically overestimates the effect their injuries had on their overall current health. A systematic review on self-assessments of pre-injury health-related QoL (Scholten et al., 2017) showed that the recalled pre-injury QoL scores of the patients consistently exceeded age- and sex-adjusted population norms. Such overestimations of past health states have been observed not only in cases involving injuries but also in association with other events such as cancer (Lindberg et al., 2017) and even dental problems (Reissmann et al., 2018) as well.

A possible explanation for these effects is *response shift*, a change in respondents’ frame of reference (Schwartz and Sprangers, 1999; Sprangers and Schwartz, 1999). A frequently used technique in response shift research is the then test (Schwartz et al., 2006). This involves respondents assessing their health at a given previous time point (t0), and another time point thereafter (t1). The respondents are then asked to assess their present health, and to retrospectively assess their health state at t0. Differences between their health as they assessed it at t0 and at t1 are generally interpreted as indicators of response shift.

However, it is also possible that the differences in ratings made at the two time points are due to a recall bias or memory effect (Blome and Augustin, 2015). Recall bias means that patients remember their previous condition differently (as being better or worse) than they felt and assessed it to be at the time. This recall bias, therefore, does not necessarily mean that respondents’ internal evaluation scale has shifted. Recall bias effects can even occur within relatively short time intervals of some weeks (Topp et al., 2019). One further possible explanation for such recall bias is the “implicit theory of change” (Ross, 1989), an idea based on the premise that individuals have an implicit concept about how their QoL has changed since the pretest, and that this concept influences their assessment of their condition at that time. This implicit theory of change can be applied not only to patients who have undergone a significant change in their state of health as a result of an illness, but also to persons in the general population. Healthy individuals also have an implicit idea of how health and QoL typically change over time, especially in older age, and this idea influences their retrospective assessments as well. Recall studies conducted among the general population can help clarify to what degree overestimations made of past health states after an event such as an injury are really due to that event, and to what degree they occur without such a “catalyst” (Ahmed et al., 2014; Schwartz et al., 2020).

Habitual *optimism* is defined as a general tendency to expect positive outcomes (Carver and Scheier, 2014; Scheier et al., 2021). Optimism is associated with positive physical (Roy et al., 2010) and mental (Carver et al., 2010) health, recovery after severe illness (Scheier et al., 1999), and even mortality (Anthony et al., 2016). Optimism also proved to be an independent prognostic factor for future health developments (Zenger et al., 2010). Per definition, optimism refers to the future and not to the present or the past. However, it remained to be systematically tested whether optimism indeed correlates more strongly with future expectations than it does with assessments applying to the present or the past. Therefore, our study also tests whether this future orientation is indeed a substantial part of the definition of optimism.

Taken together, the aims of this study were (a) To test whether people in the general population generally underrate or overrate their past (5 years before) or future (5 years later) quality of health, compared with current assessments made by people who are 5 years younger or older, (b) To analyze the correlative relationships between self-rated health for these three time points: past, present, and future, (c) To analyze the relationship between these health assessments and other variables of QoL, and (d) To test whether optimism is really related to the future more strongly than it is to the present or the past.

## Methods

### Study Participants

Data were derived from the LIFE-Adult-Study of the Leipzig Research Centre for Civilization Diseases (LIFE). This is a population-based study with a representative sample of people living in Leipzig, Germany. We obtained an age- and gender-stratified random selection of inhabitants, ranging in age from 18 to 80 years, from the local residents’ registration office. According to the study protocol, the focus was on the age range 40–80 years; the 18–39 years age range was included but underrepresented.

At the study center, the participants underwent a set of assessment batteries, including collection of their sociodemographic data, medical history, information about lifestyle factors, and several medical examinations. Pregnancy and insufficient command of the German language were the only exclusion criteria. Pregnancy was chosen as an exclusion criterion because the medical examinations might be too laborious for pregnant women. The sample was not restricted to healthy people. The participants received a lump sum of 20 EUR to cover their travel expenses. A sub-sample of the participants was asked to assess their past, present, and future health states. Details of the study design have been published elsewhere (Loeffler et al., 2015).

### Study Variables

The Visual Analog Scale (VAS) of the QoL questionnaire EQ-5D (Brooks, 1996) was used. This VAS ranges from 0 (worst possible health) to 100 (best possible health). First, the respondents were asked to assess their present health using this scale. Moreover, they were asked to assess their (recalled) health state 5 years ago and their expected health state in 5 years time, using the same 0–100 scale.

In addition to the health assessments captured with the VAS, the following questionnaires were administered: the Short Form Health Survey–8 (SF-8), for measuring QoL (Ware et al., 2001), the Generalized Anxiety Disorder screener (GAD-7), for measuring anxiety (Spitzer et al., 2006), the Multidimensional Fatigue Inventory (MFI-20), for measuring fatigue (Weis et al., 2017), the Patient Health Questionnaire-15 (PHQ-15), for measuring physical complaints (Kroenke et al., 2002), the Pittsburgh Sleep Quality Index (PSQI), for measuring sleep problems (Buysse et al., 1989), the Epworth Sleepiness Scale (ESS), for measuring daytime sleepiness (Johns, 1991), the Satisfaction with Life Scale (SWLS; Diener et al., 1985), the ENRICHD Social Support Instrument (ESSI; Berkman et al., 2003), and the Life Orientation Test-Revised (LOT-R), for measuring dispositional optimism (Scheier et al., 1994). This LOT-R comprises an optimism subscale and a pessimism subscale. The LOT-R total sum score is composed of the optimism score and the inverted pessimism score. Mean scores, standard deviations, and (if applicable) reliability coefficients in terms of Cronbach’s alpha are given in the Supplementary Table 1. Sociodemographic variables were collected by means of self-report. To calculate body mass index (BMI), body weight and height were objectively measured.

### Statistical Analysis

Associations between the health variables and the other scales were examined using Pearson correlations and partial correlations, partialing out age, sex, and SES. Socioeconomic status was calculated in accordance with the “Gesundheit in Deutschland” examination conducted by the Robert Koch Institute, Berlin (Lampert et al., 2013), integrating education, income, and professional position into one index (scoring: 1–7 for each component, and therefore, 3–21 for the total SES score). All calculations were performed with SPSS version 24.

## Results

### Sample Characteristics

For this analysis we only used the data from participants in the age range 40–74 years due to the sample sizes in the lower and the older age ranges being too small to facilitate reliable comparisons. Of the participants in this age range (40–74 years), 2282 individuals provided complete data sets including health assessments for each of the three time points. The mean age of the final sample was 53.1 years (SD = 9.7 years), and 1221 of the participants (53.1%) were females. The distribution of age groups can be inferred from Table 1. The distribution of the marital status was as follows: married and living together (57.4%), married and living separately (2.8%), never married (21.4%), divorced (15.3%), and widowed (3.1%). Regarding occupational status, the distribution was: working full time (59.5%), working part time (11.7%), unemployed (6.1%), retired (21.2%), other (1.4%). The SES mean score of the sample was *M* = 16.6 ± 3.1.

**TABLE 1:** Health assessments and their differences, broken down by age group.

Age	*n*	Present health		Past health		Future health		Diff. future-present		Diff. present-past		Diff. future-past	
		*M*	(SD)	*M*	(SD)	*M*	(SD)	*M*	(SD)	*M*	(SD)	*M*	(SD)
40–44 years	560	81.8	(14.8)	82.7	(17.8)	81.1	(14.5)	−0.7	(8.3)	−0.9	(16.2)	−1.5	(17.6)
45–49 years	510	80.6	(16.6)	82.2	(16.9)	80.4	(15.4)	−0.2	(9.8)	−1.6	(16.0)	−1.8	(16.8)
50–54 years	308	79.0	(15.8)	82.0	(16.5)	77.0	(17.2)	−2.0	(9.8)	−3.0	(15.8)	−4.9	(18.1)
55–59 years	249	75.9	(17.3)	80.6	(17.2)	72.3	(18.7)	−3.6	(11.0)	−4.6	(15.1)	−8.2	(17.9)
60–64 years	267	76.6	(16.9)	78.5	(18.3)	72.3	(18.6)	−4.3	(11.2)	−1.9	(16.4)	−6.1	(19.9)
65–69 years	226	77.7	(14.1)	79.8	(17.0)	71.0	(16.2)	−6.7	(10.1)	−2.1	(17.0)	−8.8	(17.9)
70–74 years	161	73.8	(17.4)	78.8	(16.9)	67.4	(19.1)	−6.4	(11.9)	−4.9	(16.0)	−11.4	(19.5)
Total	2282	78.9	(16.2)	81.2	(17.3)	76.4	(17.2)	−2.5	(10.2)	−2.3	(16.1)	−4.7	(18.3)

*M, mean score; SD, standard deviation.*

### Health Assessments

Table 1 presents the health assessments for the three time points and the differences between these assessments for each age group. Subjects from all age groups rated their mean past health state as having been better and projected that their future health state will be worse than their current one. These differences were small in magnitude for people younger than 50 years. The overall mean difference between past and present health states was 2.3 points, and the mean difference between present and future health states was 2.5 points (see right part of Table 1). This results in an estimated difference of 4.7 points for the total group over a 10-year period (past minus future). The difference is low in the younger age groups and increases with increasing age (Table 1).

**FIGURE 1:**
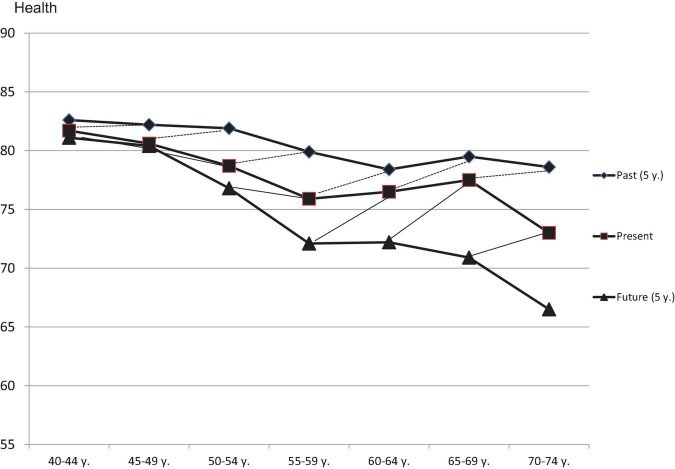
Assessments of past health (5 years ago), present health, and future health (in 5 years), broken down by age groups. The dashed and the thin solid lines connect those points that should be identical if there were no response shift effects and no recall bias effects.

Figure 1 illustrates these relationships from another perspective. Given the absence of any sampling bias, response shift and memory bias, each age group’s mean assessment of their present health state should be equal to the mean assessment the age group 5 years senior retrospectively makes of their health 5 years previously. The dotted lines in Figure 1 indicate that this is not the case; the past health states are rated as being better than they should be. If there were no effects of sampling bias, response shift, or memory bias, all of the dotted lines would be horizontal and parallel with the *x*-axis of the figure.

We observe the same pattern concerning projected future states of health. The participants expected a greater deterioration of their health than was actually experienced by the older age group based on how they rated their current health. This pattern is also more pronounced in the older age groups than in the younger groups.

### Correlations Between the Health Assessments

The correlations between the health assessments and the estimated changes in the health states are given in Table 2. The correlations between the present, past, and future health states are between 0.44 and 0.81. People who experienced a significant deterioration of their health (large difference between the present and the past score) do not expect a further significant deterioration in the future (difference between the future and the present score); the correlation is *r* = −0.10. The partial correlations did not differ significantly from the bivariate correlations (Table 2).

**TABLE 2:** Correlations and partial correlations between the health states.

	Present health	Past health	Future health	Difference future – present	Difference present – past	Difference future – past
Present health	–	0.54	0.81	–0.21	0.43	0.26
Past health	0.52	–	0.44	–0.10	–0.53	–0.53
Future health	0.81	0.42	–	0.40	0.34	0.53
Diff. future – present health	–0.26	–0.13	–0.36	–	–0.10	0.48
Diff. present – past health	0.43	–0.55	0.34	–0.11	–	0.82
Diff. future – past health	0.24	–0.56	0.51	0.45	0.83	–

*Upper right triangle: Pearson correlations; lower left triangle: partial correlations, partialization of age, sex, and SES.*

### Correlations Between Health Assessments and Other Variables

Table 3 shows the correlations between the health states, including the expected changes in health states, and other variables. Current health was negatively associated with age (*r* = −0.16) and positively associated with socioeconomic status (*r* = 0.17). There was no association between gender and health in our sample. The strongest associations between health quality and the variables in Table 3 were found for fatigue (MFI-20, *r* = −0.59), the physical component of QoL (SF-8 PCS, *r* = 0.54), and bodily complaints (PHQ-15, *r* = −0.50). The correlations between projected health quality 5 years later and the other variables were roughly as high as they were between current health and those other variables, and they were higher than those of the past health states.

**TABLE 3:** Correlations between health states and other variables.

	Present health	Past health	Future health	Diff. future – present	Diff. present – past	Diff. future – past
Age	–0.16	–0.10	–0.30	–0.24	–0.06	–0.19
Gender	–0.01	–0.08	0.05	0.09	0.08	0.13
Socioeconomic status	0.17	0.14	0.15	0.00	0.01	0.01
Education	0.07	0.05	0.09	0.05	0.01	0.04
Body Mass Index	–0.22	–0.11	–0.23	–0.02	–0.10	–0.10
PHQ-15: Bodily complaints	–0.50	–0.31	–0.43	0.06	–0.16	–0.11
GAD-7: Anxiety	–0.38	–0.26	–0.31	0.07	–0.10	–0.05
SF-8_PCS: QoL Physical comp.	0.54	0.28	0.48	–0.04	0.25	0.20
SF-8_MCS: QoL Mental comp.	0.40	0.23	0.29	–0.15	0.15	0.05
SWLS: Satisfaction with life	0.42	0.27	0.37	–0.04	0.13	0.09
ESSI: Social support	0.26	0.18	0.22	–0.04	0.07	0.04
PSQI: Sleep quality	–0.36	–0.25	–0.32	0.03	–0.09	–0.07
ESS: Daytime sleepiness	–0.10	–0.05	–0.10	–0.01	–0.05	–0.05
MFI-20: Fatigue sum score	–0.59	–0.34	–0.51	0.07	–0.23	–0.16
LOT-R: Optimism subscale	0.30	0.21	0.34	0.09	0.08	0.12
LOT-R: Pessimism subscale	–0.28	–0.18	–0.27	0.00	–0.09	–0.08
LOT-R: Optimism total score	0.36	0.24	0.37	0.06	0.11	0.13

*Optimism* (LOT-R total) was correlated with present, past, and future health (*r* between 0.24 and 0.37), cf., Table 3, bottom line. The correlations between the LOT-R total score and the present and future health were nearly identical (0.36 and 0.37), and the gain or loss in health quality expected to occur within the following 5 years was nearly independent from the LOT-R total score (*r* = 0.06).

## Discussion

The first aim of this study was to examine whether people tend to underrate or overrate the quality of health they had in the past when they assess it retrospectively. All of the age groups rated their recalled health as having been better 5 years before than the age group 5 years their junior (Figure 1) rated their current health, indicating that the first group was overestimating of how healthy they had actually been in the past. There are at least three possible explanations for this effect. The first explanation is that, due to response shift effects, the respondents changed their internal frames of reference with increasing age in the direction of tolerating more health problems and thus assessed their past health as having been relatively good. The second explanation poses the possibility of a certain memory effect. It may be that the memory stores primarily positive experiences and that the negative aspects of events experienced in the past are more often forgotten than the positive ones, leading to a positive recall of the past. The third explanation is that respondents are influenced by their own implicit theory of health change (Ross, 1989). Everyone knows that health problems increase with age, and everyone has at least a rough idea of the magnitude of this effect. It is possible that the people did not remember how healthy they were 5 years ago, and therefore inferred how much healthier they were at that point based on an internalized theory of age-related health changes. Unfortunately, we cannot decide which of these explanations are actually true. Of course, it is also possible that each of the explanations contributes to the effect to a certain degree.

We compared mean scores of one age group (present health) with mean scores of another group (past health). Such comparisons are justified only when the samples are drawn without selection bias. Compared with other studies, the decline of mean health with increasing age was low in our study. In particular, we observed a slight increase in perceived health from the group 55–59 years to the group 65–69 years. It is possible that this is indeed a selection bias. Other examinations found more continuous age gradients (Hinz et al., 2014; Huber et al., 2017). A recent Norwegian general population study, however, did not detect such an age effect at all (Bonsaksen et al., 2019). Longitudinal studies with examinations of the same people at different time points would be helpful in omitting these problems. However, even if the same respondents rated their past and current health on multiple occasions, it would still not be possible to know which of the three explanations given above actually held true, since each of these three mechanisms can affect a subject over a period of 5 years. The total mean score (*M* = 78.9) in our sample was similar to that which was obtained when the data of a representative survey of the German population (Hinz et al., 2014) were weighted according to the age distribution of our sample (*M* = 76.2).

Even if we could not determine which mechanisms explain the overestimation of the health changes best, the consequences for research using retrospective judgments are evident. In the summarizing review article (Scholten et al., 2017), it has been underlined that people who have experienced a health problem such as an injury retrospectively overrated how good their health had been prior to the injury. Our study adds that this overestimation also takes place when no such catalyst has occurred. It would be interesting to compare groups who have and have not experienced a severe health problem in the past concerning their propensity to overrate how healthy they had been beforehand.

Assessing the quality of one’s own health in the future is a speculative exercise; in doing so, subjects can only come to an estimation based on their present health and their subjective theory of future developments. The *correlation* between current health and expected health was *r* = 0.81, much higher than the past-present correlation (*r* = 0.54). This indicates that estimations of past health were derived from more than a subjective theory of change since, in that case, both correlations (present–past and present–future) would be of equal magnitude.

As was to be expected, health quality was correlated with multiple other variables: physical and mental components of QoL, life satisfaction, fatigue, bodily complaints, anxiety, sleep quality, social support, and optimism. With the exception of daytime sleepiness, all correlations were 0.26 or higher. Though it was not the primary objective of our study, the results given in Table 3 can help clarify to what degree the different components of physical and mental health contribute to how people rate their general health. For example, fatigue was more strongly associated with health (*r* = −0.59) than bodily complaints (*r* = −0.50), underlining the relevance of this symptom even in the general population.

A further topic of this study was the relationship between habitual optimism as measured with the LOT-R and how healthy people expect to be in the future. We found a positive correlation between LOT-R total scores and expected future quality of health (*r* = 0.37). However, optimistic people did not expect that their health would significantly improve in the future; the correlation between LOT-R scores and the difference between future and current health was negligible (*r* = 0.06). This means that the judgments of optimists are more positive than those of pessimists, but that this positive view is not focused solely on the future, but instead can be found to almost the same degree in the present. The correlation between future health and the LOT-R was *r* = 0.37, but the correlation with current health was nearly as high with *r* = 0.36. Though the definition of optimism concerns future events, the most often used questionnaire in the field of habitual optimism, the LOT-R, mainly assesses a person’s tendency to judge things positively irrespective of the time horizon. This seems to contradict studies which have found positive associations between optimism and future QoL outcomes even after accounting for the QoL levels of the present (Zenger et al., 2010). Future research should try to quantify which proportion of optimism is indeed related to the future and which is a general tendency to make positive assessments.

Several *limitations* of this study should be mentioned. There might be a bias in our sample. It has been shown (Enzenbach et al., 2019) that the participants of the LIFE-Adult study were somewhat healthier than the non-participants. The age dependency of the health assessment in our study was also lower than that in other studies using the same VAS (Hinz et al., 2014; Huber et al., 2017). However, the mean current health score was similar to that reported in another representative study (Hinz et al., 2014), whereas a further study (Huber et al., 2017) reported higher mean health assessments. It is possible that the inclusion of questions concerning past health influenced how respondents rated their current health as was observed in a study with patients suffering from chronic diseases (Nolte et al., 2012). We used a 0–100 VAS for rating health quality. Other options include five-point scales that measure overall health [excellent; very good, good, fair, and poor (Grol-Prokopczyk et al., 2011)] or health problems [none, mild, moderate, severe, and extreme (Guindon and Boyle, 2012)]. The advantage of the 0–100 scale is that it can be considered a metric scale which does not require non-parametric statistics. Concerning the differences between expected and real health changes, it has to be taken into account that the effect strongly depends on age. While for participants between 40 and 50 years of age the effects were small in magnitude, the age groups 60 years and older showed strong differences. We did not ask the participants in our study whether they had experienced a health-relevant event such as an injury within the last 5 years; therefore, it is possible that, unbeknownst to us, some of them had. It is unsatisfactory that we could not quantify which of the possible causes of the effects (response shift, memory effects, or subjective theory of change) contributed to the occurrence of those effects.

Despite these limitations, the results of our investigation show that comparisons between retrospectively assessed health states and current health states are problematic even in samples of the general population. Retrospective judgments are inappropriate tools for determining the impact of medical events on a person’s general state of health. Further studies which compare groups with and without health-related events are necessary to better elucidate the mechanisms underlying the changes in the assessments.

## Data Availability Statement

The raw data supporting the conclusions of this article will be made available by the authors, without undue reservation.

## Ethics Statement

The studies involving human participants were reviewed and approved by the Ethics Committee of the Medical Faculty of the Leipzig University. The patients/participants provided their written informed consent to participate in this study.

## Author Contributions

AH and SR-H designed the study. TL and SR-H were involved in data collection. AH and MF performed the statistical analyses. AH and KP wrote the first draft of the manuscript. TL, SR-H, MF, and AM-T gave feedback and contributed to the refinement of the manuscript. KP wrote the final version. All authors approved the final version.

## Conflict of Interest

The authors declare that the research was conducted in the absence of any commercial or financial relationships that could be construed as a potential conflict of interest.

## Publisher’s Note

All claims expressed in this article are solely those of the authors and do not necessarily represent those of their affiliated organizations, or those of the publisher, the editors and the reviewers. Any product that may be evaluated in this article, or claim that may be made by its manufacturer, is not guaranteed or endorsed by the publisher.

## References

[B1] Ahmed S. Sawatzky R. Levesque J.-F. Ehrmann-Feldman D. Schwartz C. E. (2014). Minimal evidence of response shift in the absence of a catalyst. *Qual. Life Res.* 23 2421–2430. 10.1007/s11136-014-0699-3 24899546

[B2] Anthony E. G. Kritz-Silverstein D. Barrett-Connor E. (2016). Optimism and mortality in older men and women: the Rancho Bernardo Study. *J. Aging Res.* 2016:5185104. 10.1155/2016/5185104 27042351 PMC4794576

[B3] Berkman L. F. Blumenthal J. Burg M. Carney R. M. Catellier D. Cowan M. J. (2003). Effects of treating depression and low perceived social support on clinical events after myocardial infarction: the Enhancing Recovery in Coronary Heart Disease Patients (ENRICHD) Randomized Trial. *JAMA* 289 3106–3116. 10.1001/jama.289.23.3106 12813116

[B4] Blome C. Augustin M. (2015). Measuring change in quality of life. Bias in prospective and retrospective evaluation. *Value Health* 18 110–115. 10.1016/j.jval.2014.10.007 25595241

[B5] Bonsaksen T. Ekeberg Ø Skogstad L. Heir T. Grimholt T. K. Lerdal A. (2019). Self-rated global health in the Norwegian general population. *Health Qual. Life Outcomes* 17:188. 10.1186/s12955-019-1258-y 31870385 PMC6929488

[B6] Brooks R. (1996). EuroQol: the current state of play. *Health Policy* 37 53–72. 10.1016/0168-8510(96)00822-6 10158943

[B7] Buysse D. J. Reynolds C. F. Monk T. H. Berman S. R. Kupfer D. J. (1989). The Pittsburgh Sleep Quality Index - A new instrument for psychiatric practice and research. *Psychiatry Res.* 28 193–213. 10.1016/0165-1781(89)90047-4 2748771

[B8] Carver C. S. Scheier M. F. (2014). Dispositional optimism. *Trends Cogn. Sci.* 18 293–299. 10.1016/j.tics.2014.02.003 24630971 PMC4061570

[B9] Carver C. S. Scheier M. F. Segerström S. C. (2010). Optimism. *Clin. Psychol. Rev.* 30 879–889. 10.1016/j.cpr.2010.01.006 20170998 PMC4161121

[B10] Diener E. Emmons R. A. Larsen R. J. Griffin S. (1985). The satisfaction with life scale. *J. Pers. Assess* 49 71–75. 16367493 10.1207/s15327752jpa4901_13

[B11] Enzenbach C. Wicklein B. Wirkner K. Loeffler M. (2019). Evaluating selection bias in a population-based cohort study with low baseline participation. The LIFE-adult-study. *BMC Med. Res. Methodol.* 19:135. 10.1186/s12874-019-0779-8 31262266 PMC6604357

[B12] Graaf M. W. D. Reininga I. H. F. Wendt K. W. Heineman E. El Moumni M. (2019). Pre-injury health status of injured patients. A prospective comparison with the Dutch population. *Qual. Life Res.* 28 649–662. 10.1007/s11136-018-2035-9 30377947 PMC6394497

[B13] Grol-Prokopczyk H. Freese J. Hauser R. M. (2011). Using anchoring vignettes to assess group differences in general self-rated health. *J. Health Soc. Behav.* 52 246–261. 10.1093/geronb/gbx048 21673148 PMC3117438

[B14] Guindon G. E. Boyle M. H. (2012). Using anchoring vignettes to assess the comparability of self-rated feelings of sadness, lowness or depression in France and Vietnam. *Int. J. Meth. Psychiatr. Res.* 21 29–40. 10.1002/mpr.1346 22411195 PMC6878381

[B15] Guyatt G. H. Feeny D. H. Patrick D. L. (1993). Measuring health-related quality of life. *Ann. Intern. Med.* 118 622–629. 10.7326/0003-4819-118-8-199304150-00009 8452328

[B16] Hinz A. Kohlmann T. Stöbel-Richter Y. Zenger M. Brähler E. (2014). The quality of life questionnaire EQ-5D-5L: psychometric properties and normative values for the general German population. *Qual. Life Res.* 23 443–447. 10.1007/s11136-013-0498-2 23921597

[B17] Huber M. B. Felix J. Vogelmann M. Leidl R. (2017). Health-related quality of life of the general German population in 2015. Results from the EQ-5D-5L. *Int. J. Environ. Res. Public Health* 14:426. 10.3390/ijerph14040426 28420153 PMC5409627

[B18] Johns M. W. (1991). A new method for measuring daytime sleepiness - the Epworth Sleepiness Scale. *Sleep* 14 540–545. 10.1093/sleep/14.6.540 1798888

[B19] Kroenke K. Spitzer R. L. Williams J. B. W. (2002). The PHQ-15: validity of a new measure for evaluating the severity of somatic symptoms. *Psychos. Med.* 64 258–266. 10.1097/00006842-200203000-00008 11914441

[B20] Lampert T. Kroll L. Mueters S. Stolzenberg H. (2013). Measurement of the socioeconomic status within the German Health Update 2009 (GEDA). *Bundesgesundheitsblatt Gesundheitsforschung Gesundheitsschutz* 56 131–143. 10.1007/s00103-012-1583-3 23275948

[B21] Lindberg P. Netter P. Koller M. Steinger B. Klinkhammer-Schalke M. (2017). Breast cancer survivors‘ recollection of their quality of life. Identifying determinants of recall bias in a longitudinal population-based trial. *PLoS One* 12:e0171519. 10.1371/journal.pone.0171519 28152108 PMC5289621

[B22] Loeffler M. Engel C. Ahnert P. Alfermann D. Arelin K. Baber R. (2015). The LIFE-Adult-Study: objectives and design of a population-based cohort study with 10,000 deeply phenotyped adults in Germany. *BMC Public Health* 15:691. 10.1186/s12889-015-1983-z 26197779 PMC4509697

[B23] Nolte S. Elsworth G. R. Sinclair A. J. Osborne R. H. (2012). The inclusion of ‘then-test’ questions in post-test questionnaires alters post-test responses. A randomized study of bias in health program evaluation. *Qual. Life Res.* 21 487–494. 10.1007/s11136-011-9952-1 21710355

[B24] Reissmann D. R. Erler A. Hirsch C. Sierwald I. Machuca C. Schierz O. (2018). Bias in retrospective assessment of perceived dental treatment effects when using the Oral Health Impact Profile. *Qual. Life Res.* 27 775–782. 10.1007/s11136-017-1725-z 29063350

[B25] Ross M. (1989). Relation of implicit theories to the construction of personal histories. *Psychol. Rev.* 96 341–357. 10.1037//0033-295X.96.2.341

[B26] Roy B. Diez-Roux A. V. Seeman T. Ranjit N. Shea S. Cushman M. (2010). Association of optimism and pessimism with inflammation and hemostasis in the Multi-Ethnic Study of Atherosclerosis (MESA). *Psychos. Med.* 72 134–140. 10.1097/PSY.0b013e3181cb981b 20100888 PMC2842951

[B27] Scheier M. F. Carver C. S. Bridges M. W. (1994). Distinguishing optimism from neuroticism (and trait anxiety, self-mastery, and self-esteem) - A reevaluation of the life orientation test. *J. Pers. Soc. Psychol.* 67 1063–1078. 10.1037/0022-3514.67.6.1063 7815302

[B28] Scheier M. F. Matthews K. A. Owens J. F. Schulz R. Bridges M. W. Magovern G. J. (1999). Optimism and rehospitalization after coronary artery bypass graft surgery. *Arch. Intern. Med.* 159 829–835. 10.1001/archinte.159.8.829 10219928

[B29] Scheier M. F. Swanson J. D. Barlow M. A. Greenhouse J. B. Wrosch C. Tindle H. A. (2021). Optimism versus pessimism as predictors of physical health: a comprehensive reanalysis of dispositional optimism research. *Am. Psychol.* 76 529–548. 10.1037/amp0000666 32969677

[B30] Scholten A. C. Haagsma J. A. Steyerberg E. W. van Beeck E. F. Polinder S. (2017). Assessment of pre-injury health-related quality of life. A systematic review. *Popul. Health Metr.* 15:10. 10.1186/s12963-017-0127-3 28288648 PMC5348891

[B31] Schwartz C. E. Bode R. Repucci N. Becker J. Sprangers M. A. G. Fayers P. M. (2006). The clinical significance of adaptation to changing health: a meta-analysis of response shift. *Qual. Life Res.* 15 1533–1550. 10.1007/s11136-006-0025-9 17031503

[B32] Schwartz C. E. Sprangers M. A. G. (1999). Methodological approaches for assessing response shift in longitudinal health-related quality-of-life research. *Soc. Sci. Med.* 48 1531–1548. 10.1016/S0277-9536(99)00047-7 10400255

[B33] Schwartz C. E. Stucky B. D. Michael W. Rapkin B. D. (2020). Does response shift impact interpretation of change even among scales developed using item response theory? *J. Pat Rep. Outcomes* 4:8. 10.1186/s41687-019-0162-x 31975159 PMC6977794

[B34] Spitzer R. L. Kroenke K. Williams J. B. W. Lowe B. (2006). A brief measure for assessing generalized anxiety disorder - The GAD-7. *Arch. Intern. Med.* 166 1092–1097. 10.1001/archinte.166.10.1092 16717171

[B35] Sprangers M. A. G. Schwartz C. E. (1999). Integrating response shift into health-related quality of life research: a theoretical model. *Soc. Sci. Med.* 48 1507–1515. 10.1016/S0277-9536(99)00045-3 10400253

[B36] Tengs T. O. Wallace A. (2000). One thousand health-related quality-of-life estimates. *Med. Care* 38 583–637. 10.1097/00005650-200006000-00004 10843310

[B37] Topp J. Andrees V. Heesen C. Augustin M. Blome C. (2019). Recall of health-related quality of life: how does memory affect the SF-6D in patients with psoriasis or multiple sclerosis? A prospective observational study in Germany. *BMJ Open* 9:e032859. 10.1136/bmjopen-2019-032859 31753898 PMC6887080

[B38] Ware J. E. Kosinski M. Dewey J. E. Gandek B. (2001). *How to Score and Interpret Single-Item Health Status Measures: A Manual for Users of the SF-8TM Health Survey.* Lincoln: QualityMetric Incorporated.

[B39] Weis J. Tomaszewski K. A. Hammerlid E. Arraras J. I. Conroy T. Lanceley A. (2017). International psychometric validation of an EORTC quality of life module measuring cancer related fatigue (EORTC QLQ-FA12). *J. Natl. Cancer Inst.* 109:djw273. 10.1093/jnci/djw273 28376231

[B40] Zenger M. Brix C. Borowski J. Stolzenburg J. Hinz A. (2010). The impact of optimism on anxiety, depression and quality of life in urogenital cancer patients. *Psycho Oncol.* 19 879–886. 10.1002/pon.1635 19862795

