# Supplementary material for: Relationships Between Self-Rated Health at Three Time Points: Past, Present, Future
Source: Front Psychol. 2022 Jan 13;12:763158. doi: 10.3389/fpsyg.2021.763158 (PMC8793032; doi:10.3389/fpsyg.2021.763158)
Supplement: Supplementary file 1 [file Table_1.pdf]

Supplementary TABLE S1 Mean scores, standard deviations, and Cronbach's alpha coefficients (if applicable) of the additional variables

	M	SD	Alpha
PHQ-15: Bodily complaints	5.24	3.82	.77
GAD-7: Anxiety	3.51	3.37	.85
SF-8_PCS: QoL Physical comp.	48.79	8.15	-
SF-8_MCS: QoL Mental comp.	52.23	8.57	-
SWLS: Satisfaction with life	26.47	5.59	.90
ESSI: Social support	22.27	3.58	.92
PSQI: Sleep quality	4.78	3.26	-
ESS: Daytime sleepiness	8.29	3.60	.71
MFI-20: Fatigue sum score	40.53	13.53	.92
LOT-R: Optimism subscale	8.97	2.40	.68
LOT-R: Pessimism subscale	4.35	2.36	.63
LOT-R: Optimism total score	16.62	3.83	.68
